# Automatic Number Plate Recognition:A Detailed Survey of Relevant Algorithms

**DOI:** 10.3390/s21093028

**Published:** 2021-04-26

**Authors:** Lubna, Naveed Mufti, Syed Afaq Ali Shah

**Affiliations:** 1 Department of Telecommunication Engineering, University of Engineering and Technology, Peshawar 25000, Pakistan; Lubnaxafi@gmail.com; 2 Department of Telecommunication Engineering, University of Engineering and Technology, Mardan 23200, Pakistan; 3 Department of Information Technology, Media and Communications, Murdoch University, Murdoch 6150, Australia; Afaq.Shah@murdoch.edu.au

**Keywords:** automatic number plate recognition, image processing, computer vision, machine learning, vehicle identification, neural networks, intelligent transportation system, smart vehicle technologies, object detection and tracking, recognition

## Abstract

Technologies and services towards smart-vehicles and Intelligent-Transportation-Systems (ITS), continues to revolutionize many aspects of human life. This paper presents a detailed survey of current techniques and advancements in Automatic-Number-Plate-Recognition (ANPR) systems, with a comprehensive performance comparison of various real-time tested and simulated algorithms, including those involving computer vision (CV). ANPR technology has the ability to detect and recognize vehicles by their number-plates using recognition techniques. Even with the best algorithms, a successful ANPR system deployment may require additional hardware to maximize its accuracy. The number plate condition, non-standardized formats, complex scenes, camera quality, camera mount position, tolerance to distortion, motion-blur, contrast problems, reflections, processing and memory limitations, environmental conditions, indoor/outdoor or day/night shots, software-tools or other hardware-based constraint may undermine its performance. This inconsistency, challenging environments and other complexities make ANPR an interesting field for researchers. The Internet-of-Things is beginning to shape future of many industries and is paving new ways for ITS. ANPR can be well utilized by integrating with RFID-systems, GPS, Android platforms and other similar technologies. Deep-Learning techniques are widely utilized in CV field for better detection rates. This research aims to advance the state-of-knowledge in ITS (ANPR) built on CV algorithms; by citing relevant prior work, analyzing and presenting a survey of extraction, segmentation and recognition techniques whilst providing guidelines on future trends in this area.

## 1. Introduction

Automatic Number Plate Recognition has become part of our lives and promises to stay in future, integrable with proposed transportation technologies. The concept of Autonomous Vehicles is offering many possibilities of changing fundamental transportation systems. ANPR technology is already contributing towards intelligent transportation systems and is eliminating the need of human intervention. It is no longer just the camera on the roadside or at the barrier to the car park. It has become over the years mobile, first being deployed in vehicles, but now more recently with the advent of smart phone technology, many ANPR systems have become handheld too. Due to lower provisioning costs, ANPR is often a choice in the toll and parking lot businesses. The main reason is that the ANPR system recognizes the registered number plate with no additional transponder requirements, as compared to the Ultra High Frequency—Radio Frequency Identification (UHF-RFID) systems. The rapid urbanization of countries is a great advancement in our modern world. People migrate away from rural areas and choose to live in cities mostly. Local governments often fail to recognize the present and potential mobility needs of residents and visitors as traffic rises in these areas. ANPR is being increasingly used to examine the free flow of traffic, facilitating the intelligent transportation [1].

**Figure 1:**
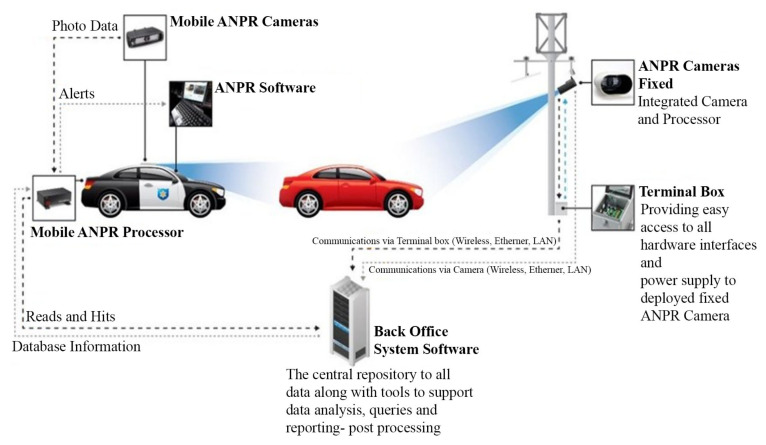
Typical ANPR System Diagram of a Fixed ANPR System (**right**) and a Mobile ANPR System (**left**) (Source: latech.us, accessed on 5 November 2020).

Not only can modern ANPR cameras read plates, but they can provide useful additional information such as counting, direction, groups of vehicles and their speed. The ability to detect and read large volumes of fast moving vehicles has meant that ANPR technology has found its way into many aspects of today’s digital landscape. Whilst ANPR technology can come in many different packages, they all perform the same basic function which is to provide a highly accurate system of reading a vehicle without human intervention. It is utilized in very diverse applications such as access control, parking management, tolling, user billing, delivery tracking, traffic management, policing and security services, customer services and directions, the red light and lane enforcement, queue length estimation, and many other services [2,3,4,5,6,7,8]. Figure 1 shows the basic system diagram of a fixed and mobile ANPR technology.

**Figure 2:**
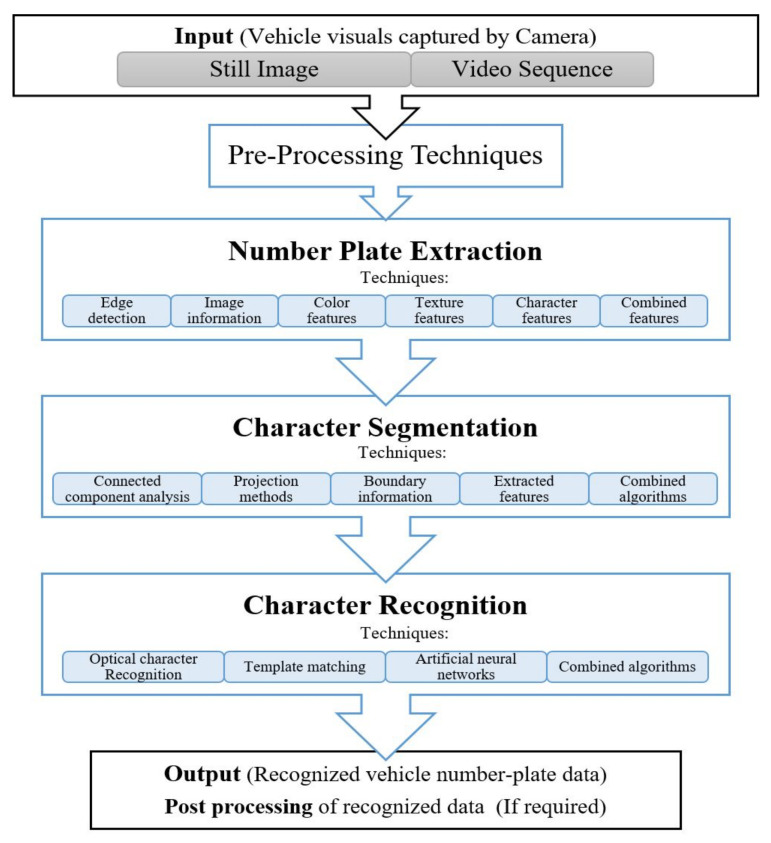
General processes of number plate recognition system.

Number Plate Recognition involves acquisition of number plate images from the intended scene, using a camera. Either still images or a photographic video is captured and further processed by a series of image processing based recognition algorithms to attain an alpha-numeric conversion of the captured images into a text entry. After obtaining a good quality image of the scene/vehicle, then the core dependence of any ANPR system is on the robustness of its algorithms. These algorithms need a very careful consideration and require thousands of lines of software coding to get desired results and cover all system complexities. As a whole, a series of primary algorithms are necessary for smart vehicle technologies and ANPR to be effective. The general processes involved in ANPR systems is shown in Figure 2.

A typical ANPR system goes through the general process of image acquisition (input to the system), number plate extraction (NPE), character segmentation (CS) and character recognition (CR) (as output from the system) [9]. After successful recognition of the vehicle the data can be accessed and used for post processing operations as required. The vehicles data is sent to the connected back office system software which is the central repository to all data along with tools to support data analysis, queries and reporting accordingly. This data collected can be utilized for several other intelligent transportation applications since ANPR systems not just visually capture the vehicle images but also record the metadata in their central repository. This can potentially include vehicle recognition through date and time stamping as well as exact location, whilst storing a comprehensive database of traffic movement. This data can be helpful in modelling different transport systems and their analysis.

**Figure 3:**
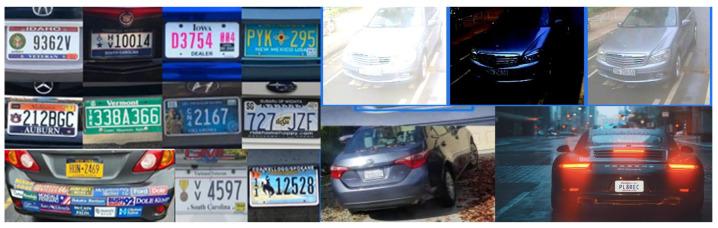
License plate diversity in styles, colors, fonts, sizes, and physical conditions. (Source: Plate Recognizer ALPR [14]-a division of ParkPow [15]).

The image taken from the scene may experience some complexities depending upon the type of camera used, its resolution, lightening/illumination aids, the mounting position, area/lanes coverage capability, complex scenes, shutter speed and other environmental and system constraints. Figure 3 shows License plate diversity in styles, colors, fonts, sizes, and physical conditions; which may affect the recognition accuracy. When a vehicle is detected in the scene/image, the system uses plate localization functions to extract the license plate from the vehicle image, a process commonly termed as Number Plate Extraction. Characters on the extracted number plate are then segmented prior to recognition process. Character segmentation is an algorithm that locates the alpha numeric characters on a number plate. The segmented characters are then translated into an alpha numeric text entry using the optical character recognition (OCR) techniques. For character recognition, algorithms such as template matching or neural network classifiers are used. The performance of an ANPR system relies on the effectiveness of each individual stage. A parameter used to quantify the whole process is the performance-rate or success-rate, which is the ratio of the number of number-plates successfully recognized to the total number of input images taken. The performance rate involves all the three stages of recognition process, number plate extraction, segmentation and character recognition.

The ANPR system collects the primary form of the information from ANPR software including the images and its associated metadata. It provides the transport system with automation and security features. Its integration in ITS makes it possible to automate the system by providing services in toll collections, traffic analysis, improving law enforcement’s and building a comprehensive database of traffic movements. Integrating ANPR with Information Communication Technology (ICT) tools is another useful feature of the technology. The data from ANPR systems can be well utilized for modelling and implementation of various aspects of transport systems such as to model Passenger Mobility systems [10], traffic flow analysis and road network control strategies using Network Fundamental Diagram (NFD) models [11], in vehicle routing choice model to decide on Route and Path Choices of Freight Vehicles [12] and travel demand patterns through Floating Car Data (FCD) [13].”

There are different terminologies used for ANPR systems:

- Number plate Recognition (NPR)
- Automatic License Plate Recognition (ALPR)
- License Plate Recognition (LPR)
- License Plate Recognition (LPR)
- Automatic Vehicle Identification (AVI)
- Car Plate Recognition (CPR)

This paper provides a systematic review of the existing ANPR techniques. It covers the main features of ANPR systems by analyzing their performance summary, pros and cons accordingly. This research aims to advance the state of knowledge in smart vehicle technologies for future researchers by:

- Providing a detailed knowledge on past and current algorithms for Automatic number plate recognition systems
- Analyzing and presenting a survey of ANPR image processing based techniques for each stage systematically, (Number plate: Extraction, Segmentation followed by Recognition), with relevant brief of the techniques used at each stage along with the performance summary, where applicable
- Summarizing the performance of different algorithms used and tested by various researchers for vehicle recognition
- Summarizing previous reviews and surveys related to ANPR, as in Table 1
- Providing the performance summary with analysis and limitations, as in Table 2
- Providing list of useful ANPR datasets which the researchers may utilize to test their algorithms, as in Table 3
- Discussing current and future trends in the area of ANPR
- Discussing and citing relevant prior work for all ANPR techniques listed

Number plate extraction methods are categorized in Section 2. Number plate Segmentation and Recognition Methods are discussed in Section 3 and Section 4, respectively. Discussion is provided in Section 5. Conclusion and future research directions are presented in Section 6.

Table 1 summarizes previous reviews and surveys related to ANPR [16,17,18,19]. Table 2 presents performance summary of ANPR system techniques considering their relevant performance parameters. Table 3 enlists some of the available ANPR datasets to the research community.

## 2. Number Plate Extraction Methods

Successful extraction of number plate from the image/video is initially the most important and critical stage for ANPR systems. The extraction rate is the rate of successfully extracted plates to the total number of input images or vehicles detected from the scene. Typically, a single camera is installed on each lane and some advanced cameras may allow multiple lane support given their high resolutions. Multiple lanes installation requires multiple readers to identify vehicle number plates and more hardware with higher costs to service and maintain. Real time scenarios may face multiple challenges. For instance, the camera installed at a fixed position may acquire images of the vehicle with tilted or skewed number plate characters. It is possible that number plate is obscured with dirt or broken or located at position that is out of sight to the camera (since different types of vehicles have their number plates affixed at different positions of the vehicle body). Environmental factors, light, motion blur, reflections, fog, and other similar conditions makes it challenging for the system to extract the number plate efficiently. Algorithms using geometrical features for extracting the rectangular shaped number plates may have issues if there are multiple similar shapes drawn/pasted over the car body. Along with rectangular shaped features, further algorithms must be used to eliminate the unwanted regions.

The algorithms need to be robust to differentiate between the number plate and other objects in the image frame. Researchers have used various features for the extraction of number plates. A brief study of these feature extraction algorithms is presented in the following section.

### 2.1. NP Extraction Using Edge Information

The number plate typically has a known aspect ratio and a rectangular shape. In [19], the images were first scaled to a fixed aspect ratio. The authors evaluated and put to test various algorithms from the past proposed research in [20,21,22] and compared the results by implementing it for their own dataset. One of the number plate extraction methods they evaluated is based on the vertical edge information. It detects the vertical edges using Sobel operator. The number plate is localized by comparing the preset minimum and maximum lengths with that of the extracted edges and removing the unwanted ones. The total extraction rate for 141 images is 65.25% which is lower than the 99.99% originally reported in [20]. In [23], vertical and horizontal edge histogram information is used for number plate extraction. Testing 50 images of various fonts and light conditions resulted in 90% extraction accuracy.

Edge detection algorithms are commonly used to extract number plates by finding all the rectangles in the acquired images [24,25,26]. Mostly, the car body and number plate area have a clear color transition. The differentiation between the two is done by identifying the edges using edge detection filters or algorithms. Sobel filter, a simple algorithm, has been used for the successful edge extraction as presented in [19,20,27,28]. Edges are detected by performing vertical edge detection to extract the vertical lines and horizontal edge detection to get the horizontal lines or simultaneous use of both to extract a complete rectangular shape. The number plate can be detected by using the geometric attributes by locating the rectangle lines. Various edge detection filters Sobel, Canny, Gabor & Log-Gabor filters for ANPR systems are compared in [29].

Gabor filters are considered the best choice for structure recognition as they show exceptionally good results for excluding clamor/noise while saving edges [30].

In [20,27,31], the indented number plate region is extracted by using the magnitude of vertical edges, and this is considered as the most robust extraction feature. The vertical edges are compared to acquire the intended rectangles which are then filtered for the one rectangle being the number plate area using the known aspect ratio. In [20], it is stated that if the background edges are removed and the vertical edges are obtained, the number plate can be successfully extracted from the remaining edges in the image. The total processing time for an image of size 384 × 288 is 47.9 ms and the detection rate was about 100% for 1165 test images.

Vertical Edge Detection Algorithm (VEDA) is said to be the robust algorithm for edge detection as proposed for number plate extraction in [32]. The extraction rate for 50 images in various lighting conditions is 96%. The horizontal edges can result in possible errors. These errors occur mainly due to the car bumper [33]. From the literature, we can also find the block-based method for this purpose. According to [34], the possible number plate areas are the blocks having the high edge magnitude. This method is independent of the boundary edges of the number plate and can be applied to identify it from unclear images too. An accuracy of 92.5% is obtained for character recognition, using a pair of 180 different images. In [35], tests were carried out to check the inspection status of motorcycles by recognizing its number plate. A success rate of 95.7% for roadside and 93.7% for inspection stations test images was achieved. In [36], Hough Transform is used to extract the number plate using the boundaries. The number plate is located by detecting straight lines in the test image. This transform has the ability to detect straight line with an inclination of up to 30 degrees. However, it is computationally expensive and requires large memory. In [37], the generalized symmetry transform is used. The corners from the edges in the image are detected by scanning them in selective directions. By using the generalized symmetry transform the number plate region is extracted by detecting the similarities between these corners. The continuity of the edges is important when using the edge based methods as these are considered to be simple and fast. The extraction rate can significantly improve by eliminating the unwanted edges using some morphological steps. A combination of morphology and the edge statistics was proposed in [38]. Prior to this, the basic pre-processing techniques were also applied to enhance the image for color contrast and noise removal. 98% of successful extraction and 75–85% overall performance rate is achieved by testing on 9745 images.

### 2.2. NP Extraction Using Global Image Information

In binary image processing, the image is scanned and its pixels are labeled into components based on the pixel connectivity using the Connected Component Analysis (CCA) technique [39,40,41]. In [42,43], for extraction of number plate, the spatial measurements are used which are commonly the aspect ratio and area.

Slimani et al. [39] proposed a two-step method for the number plate extraction. To cope with the variant light illumination conditions, the first step uses Otsu’s Threshold Method which is an efficient and simple method for adaptive thresholding techniques. The binarized image is then detected for rectangular shapes in it using the CCA technique. The second step applied to the obtained number plate is to perform the edge detection followed by closed curve method to make sure that the generated image is a number plate. Over 2500 Moroccan format images from video sequences were tested using this method with a success rate of 96%. The connected component analysis technique used in [43] recorded a successful extraction rate of 96.6% on a low-quality video with a length of over four hours. Contour detection techniques are used on binary images to locate the connected objects [44]. The geometrical features having similarity with the number plate is chosen for further processing. However, this algorithm may result in distortion errors if the acquired image is of low quality.

In [45], cross correlation is used for the same task. To extract the number plate region, a pre stored number plate template is used while performing the 2D cross correlation, making it independent of its position in the image. However, it is considered to be a time-consuming method.

### 2.3. NP Extraction Using Color Features

Vehicle number plates can be color specific for some regions or countries. As we study the work reported till date, color based extraction of number plate from the image is also tested for ANPR systems.

General approach of number plate extraction involves the idea of color combination of the number plates. Also, the characteristics are unique, and such color contrasts take place solely in a plate region. Shi et al. [46] proposed a technique for the specific patterns of Chinese number plates. Their technique employed all pixels from the acquired image and then grouped in terms of hue, lightness, and saturation (HLS). The HLS color model classifies the pixels into 13 color categories instead of 6 RGB divisions. Color model was used instead of grayscale. The color division selection is made according to China Mainland number plate formats. In their experiment, 90% of the total images were correctly recognized in various illuminations.

In [47], recognition of only certain colors including black, green, white, and red-are used in number plates, color edge detectors work only on three types of colored edges including red-white, black-white, and green-white combinations. While experimenting under different conditions, about 1088 images were analyzed from various scenarios. The localization rate of number color plate is 97.9%. In [48], to classify each pixel color, a neural network is utilized after transforming the true color image into hue, saturation, lightness (HLS) color model. Neural network outputs the white-green-red colors being the specific colors used in Korean number plates. To detect the region of highest color density, the same color combination is projected both horizontally and vertically in the number color plate.

To create an edge image, the combination of character color as well as number plate color is used in [49]. The image generated is then scanned horizontally. In case any pixel having value within the range of the number color plate exists, the color range of its horizontal neighbors is examined. If minimum two or more than two horizontal neighbors belong to the same range, then that pixel is termed as the edge pixel. Eventually, all the edges are analyzed in the new edge image, which is done to determine the color plate regions.

In [50,51], mean shift algorithm is applied for segmenting the color images into candidate regions. Later on, these candidate regions are labeled as either a number plate area or discarded. A detection rate of 97.6% accuracy was recorded. In [52], another algorithm namely, a fast-mean-shift method was proposed. Mean Shift method is widely used in feature analysis including image and video segmentation. The authors utilized the feature of segmenting the complex background into possible segments and thus extracting the candidate number plate region. A database of 400 images, captured under various light conditions having 640 × 480 pixels size, were tested using the proposed method. A detection accuracy of 92.6% was achieved.

To deal with the varying illuminations problems, [53] came up with an algorithm based on fuzzy logic. The HSV color space is utilized in the process. In the fuzzy sets, all the components of HSV are initially mapped in terms of the different membership-functions. Then the fuzzy classified function is demonstrated by fusing all the three HSV components based weighted membership degrees. Three different image datasets were chosen for testing the efficiency. The recognition rate on average as recorded is 95.05% on Shanghai, 92.17% on Shenzhen and 93.23% on Beijing number plates respectively. In [54], to determine candidate regions, the statistical threshold is chosen by the adopted color model of HSI, where HSI represents Hue, Saturation, and Intensity. This technique can be utilized to detect candidate regions provided the number plates and the car/vehicle bodies are of the same color. The standard deviation and mean of hue are applied in the detection of yellow and green pixels of number plate. The intensity and saturation component of HSI are utilized for sending the white, green, and yellow pixels of number plate from the relevant vehicle images.

There is an advantage of applying number plate extraction method by using color information. That is, you get to determine the number plates that are either deformed or inclined. However, it comes with some drawbacks as well. It is challenging to define the color of the pixel using the RGB value, particularly under specific conditions of illumination. The HLS color model [48], which is used as a substitute, is quite noise sensitive. Methods that work on color projection deal with the drawback of incorrect detection of the image parts that have the exact color on the number plate as the car body.

### 2.4. NP Extraction Using Texture Features

These methods are dependent on the characters in the number plate area. A grey scale shows a significant change due to color transition with a high edge density between the character colors on the plate and its background.

Local Binary Pattern (LBP) [55] and Histogram of Oriented Gradients (HOG) [56] are used in [36], for number plate extraction. Considering the rectangular shape of the number plate, along HOG, the LBP algorithm is used for classifying the texture and calculating a histogram. The accuracy of locating 110 images is 89.7%. However, this technique is not useful for images with blur or low light conditions and different angle orientations.

Another effective technique for plate detection used by many researchers is to utilize the line weight density map. This can be combined with other techniques to improve results. In [49,57,58,59,60,61,62,63], the scan line technique is utilized where the peaks are formed from the color transition of the grey scale level which corresponds to the number of characters on the number plate. In [57], the authors proposed the horizontal line scanning with multiple thresholds technique for plate detection in a real time/complex images. The experimental results were compared to the conventional model based on Hough transforms with low detection accuracy of 69.8% and longer processing time of 8–10s, as in [58]. In comparison to [58], the extraction rate of 99.2% was achieved using line scan technique in [57]. The execution time for locating the plate was incredibly reduced to 0.3–0.5s comparatively.

In [59], the edge lines were selected using a weight density map. This technique was implemented on a certain set of images and the results were effective with an extraction rate of 93%, whereas for different image standards the effectiveness dropped to 83%. In [60], the weight density map is combined with neural network based algorithms. A dataset of 400 varying conditions images were tested. This hybrid approach proved to be effective, with a success rate of 97.23% and average extraction time of 0.0093 s per image, based on simulations results. In [49], the authors used histogram data with combined features of morphology. 97.7% success rate is recorded for 360 images set under different conditions. Vector quantization technique is used to detect the characters in the image by mapping the higher contrast regions into smaller blocks. Using different quality images, it yielded 98% of successful detection with a processing time of 200 ms [64].

The use of sliding concentric window was proposed in [54,65]. It uses the texture irregularities in the image. The area with abrupt changes is considered to be the candidate number plate region. In [54], histogram was used along with the sliding concentric window method. Texture analysis is widely used for number plate extraction. Gabor filters being the candidate tools had the ability to analyze textures in any number of orientation and scales [66]. It is, however, a computationally expensive method. While using fixed and specific angled images, it resulted in a 98% success rate [67].

Wavelet transform is used in [62,68]. In [62], horizontal reference lines with wavelet transform processing achieved a success of 92.4%. In [68], using wavelet transform, an accuracy of 97.3% is achieved with a processing time of 0.2 s. A combination of Haar-like features and adaptive boosting is used in [69,70]. This feature is commonly used to detect objects and is invariant to the position, size, contrast or color of the number plate. The gradient density is used by the cascade classifiers in [69], with a detection success of 93.5%. With adaptive boosting technique, images of different sizes, formats and with various illumination conditions, yielded 99% detection rate [71].

Texture based techniques are independent of the number plate boundary and can detect number plate properly even if it is deformed. However, these techniques are computationally complex to extract number plate from such images, if too many edges are detected in the image or the background has multiple elements, or the light illumination is not enough.

### 2.5. NP Extraction Using Character Features

Techniques based on character feature extraction are also proposed in various research studies, which are associated with locating the plate’s characters. These methods scan the image for characters on the number plate, if any. The region where the relevant characters are identified, is then extracted as number plate region. In [72], the algorithm identifies all the character areas from the image rather than directly applying the number plate properties. This process is executed by using the character-like region dependent approach. All the identified character-based regions are mentioned and classified using neural network. In [73], the aspect ratio of binary image objects is the same as that of the characters and around 30 image pixels are labeled. Hough transformation is used to identify the straight lines. The same transform is applied to both upper and lower parts of these binary labeled objects. When two lines are parallel to each other and lie within a specified range and has the same number of objects as the characters, the region between these lines is termed as the number plate area. In [74], the scale-space analysis is used to extract the number plate characters. This technique extracts blob type large sized figures that include line type of smaller figures as character candidates. In [75], firstly, the regions containing characters are identified in terms of the character width and the difference between character region and its background. Then the number plate extraction process is executed in the plate region to identify inter-character distance. This extraction technique produces an extraction accuracy of about 99.5%. In [76], the first stage character classifier obtains a primary set of all the possible character-like regions. Then the set is passed to the next stage classifier. This second stage classifier process eliminates the non-character regions from the initial set. In this technique, 36 AdaBoost classifiers act as primary stage classifier. The second stage classifier is employed with Support Vector Machine (SVM), which uses Scale-Invariant Feature Transform (SIFT) algorithm, to detect and describe local features of number plate images. These techniques of feature extraction from binary images, to define the number plate region, take quite long time. The reason is attributed to the processing of all the objects from those binary images. These processes also generate errors if the image contains other texts on it.

### 2.6. NP Extraction Using Feature Learning

For effective detection of number plate, few extraction methods look for at least two or more characteristics of the number plate. In this case, the extraction techniques are considered as hybrid extraction methods [77].

You Only Look Once (YOLO), a Convolutional Neural Network (CNN) based object detector, has been utilized in [78]. It is a two-step method that employs easy data augmentation strategies like flipped characters and inverted number plates. The CNNs are fine-tuned and trained at each stage. The resulting model yields good results for two distant datasets. The first database SSIG-SegPlate Database by Smart Surveillance Interest Group (SSIG), includes 2000 frames from about 101 vehicle videos. The system achieves 93.53% recognition accuracy at 47 FPS, functioning better than both the commercial systems of OpenALPR and Sighthound (with respective recognition rates of 93.03% and 89.80%), and substantially better results than the past techniques [79,80], which achieve accuracy of 81.80%. The other dataset used has varying image conditions, similar to real time scenarios. This public database is termed as UFPR-ALPR. The UFPR-ALPR dataset is the property of the Laboratory of Vision, Robotics and Imaging (VRI) at the Federal University of Paraná, Brazil) dataset. It contains around 4500 frames and 150 videos taken while both the vehicles and camera are in motion. The dataset contains several types of vehicles including buses, motorcycles, cars, and trucks. The test versions of commercial systems yielded the rate of recognition which is less than 70% while this system functions well with 35 FPS and identification rate of 78.33%. Bulan et al. [81], attained greater accuracy in NP recognition adjacently implementing the character recognition and segmentation with Hidden Markov Models (HMMs). Here, the most probable NP was decided by running the Viterbi algorithm.

Extraction of texture and color features are combined in [82,83,84,85,86]. In [82], rules from fuzzy logic method are used to extract yellow colors and texture feature. The color values of yellow, obtained from the demo images, are employed to train color feature fuzzy classifier. This fuzzy classifier is trained according to the color transition between number plate background and characters. Each of the pixels is classified, for any given image, if it belongs to the fuzzy rules generated number plate. In [83], two neural networks are employed for determining the color and texture characteristics. One of the neural networks is trained for texture detection using several edges within the plate area. The other is employed for color detection. These neural networks’ outputs are used in identifying the candidate regions.

In [84], there is a single neural network utilized for image scanning with H × W window, which resembles the size of vehicle plate. This network is used to sense the edges and color within this window to determine if it is a candidate area containing number plate. In [85], the neural network horizontally scans an HLS image with a 1 × M window. Here, M represents an approximate width value of the number plate, and vertical scanning is done with an N × 1 window, where N indicates the height of the plate. For every pixel of the image, the hue value is used to denote the color details and intensity value represents the texture details. Resultant of both the horizontal and vertical scans is combined to extract the candidate areas of the plate. In [86], Time-Delay Neural Network (TDNN) is processed for number plate extraction. Two such TDNNs are implemented in the color and texture analysis of the number plate by checking small windows of the image’s horizontal and vertical cross sections. Based on pixel values similarity with the number plate, the area with higher edge density is extracted as a number plate. Edge and color information based approach is used for extraction in [87]. Covariance matrix has been used for plate extraction in [88]. It is based on combining the spatial information and statistical data. Each matrix has adequate amount of information and it is enough to match the area in multiple views. This matrix is used to train the neural network efficiently in order to detect the number plate area. A combination of texture shape and color features is used in [89]. The number plate extraction from 1176 images shows extraction rate of 97.3%, considering various light illumination condition and scenes. Connected Component Labelling (CCL), threshold and Gabor filter are combined in [66] to extract number plates. To detect edges, wavelet transform is used in [87]. This is done by employing the morphology techniques once edges are detected. The shape and structure information analyzed from the input images is helpful to localize the number plate. HLS color decomposition, Hough detection and wavelet analysis are proposed in [90]. In [91], two-dimensional Discrete Wavelet Transform (DWT) is used. The proposed method successfully eliminates the background noise by highlighting the number plates’ vertical edges. The plate extraction was done using orthogonal projection histogram inspection and Otsu’s segmentation. The most accurate candidate is then chosen on the basis of edge density verification and aspect ratio constraint. In [92], the number plate detection is done by Modified Census Transform (MCT) computed local structure patterns. After that, two post-processing parts are utilized to reduce the incorrect positive rates. One post-processing includes the position-based technique between a vehicle plate and a false positive that has indifferent local structure patterns like radiators or headlights. The other one is the color-dependent method that utilizes entire color details of the number plates. Deep Learning (DL) techniques based ANPR systems generally address the character identification and segmentation as a whole. Montazzolli et al. [93] proposed a CNN architecture for character segmentation and recognition. The experiment was carried out using a publicly available dataset. In their technique, 99% of the characters were segmented successfully while the accuracy of reading the segmented characters was 93%. However, in spite of the outstanding achievements of DL techniques in ANPR [79,94,95], ANPR datasets with cars/vehicles and NPs annotations still have a huge demand. The training data set is responsible for the progressive performance of DL methods. A huge training data will help in the training of data hungry deep neural networks and better utilization of more robust network architectures along with additional layers and parameters.

## 3. Number Plate Segmentation Methods

The character segmentation stage is totally dependent on the success of number plate extraction from the image or scene. The isolated number plate may experience issues such as contrast problems, varying illumination conditions, or it may be oriented at variable angles. In such cases, pre-processing techniques, de-skewing, de-blurring or any other methods, depending upon the conditions of the number plate, may need to be applied before segmenting the characters. This step is carried out either at the extraction stage or after getting an isolated candidate area, which depends on the approach followed. To deal with tilted number plate images, preprocessing technique like bilinear transformation is used in [48,96], the isolated number plate is mapped onto a straight rectangular shape. Tilted number plates are fixed using a least square method in [97], it treats both the vertical and horizontal oriented tilts. In [98], using the Karhunen-Loeve (K-L) transformation, the character coordinates are organized into 2D variance matrix. After that, the angle of rotation and Eigenvectors are computed, and tilt correction is implemented for both vertical and horizontal tilt of the image. Three techniques namely: K-means cluster based line fitting, least squares based line fittings, and K-L transform are suggested to compute the angle of vertical tilt. Threshold application seems simple while converting to binary image, but it is a very challenging part in the whole process. An inappropriate threshold value may result in connected characters, with either within characters or with the number plate frame, which makes it difficult for segmentation [97]. A single value threshold may not be appropriate, for all the images, due to variance in image conditions and lighting. Image enhancement is necessary before binarizing the image. Enhancement includes noise removal from the image, enhancing its contrast or to apply histogram equalization techniques. In [99], to implement gradient analysis over the entire image, a technique was proposed to sense the number plate followed by the enhancement of the plate by grey color transformation. In [100], the Niblack binarization algorithm adjusts the image threshold according to the standard deviation and local mean. In [101], for every pixel, local threshold technique is applied. The threshold value is obtained by subtracting the specified constant value from the average grey levels in an m×n window placed in the middle of the pixel. In [102], a new method was proposed to reduce noise and characters enhancement. The character size was assumed to be about 20% of the size of number plate. Initially, the level of the grey scale is ranged between 0–100. Then 20% larger pixels are scaled by a factor of 2.55. The noise pixels are minimized whereas the characters are intensified. As the image binarization is unable to generate desirable results with one global threshold, method of adaptive local binarization is followed.

In the following, number plate segmentation methods are reviewed, based on the features used.

### 3.1. NP Segmentation Using Connected Components

Pixel connectivity is used for segmentation in [23,24,26,27,45,47,103]. In [26], 958 High Definition (HD) images with various conditions are tested using connected component labeling with 99.75% segmentation accuracy. The character pixels in the binary image are labeled based on their connectivity and are analyzed for aspect ratio and size with that of the number plate characters. However, this method does not seem promising for joined characters or broken ones. In [23], a limited dataset of 50 images were tested using connected component labeling and morphological method, achieving a segmentation rate of 91%. In [24], using connected components analysis, an accuracy rate of 99.5% was recorded by employing HD images in various light and weather conditions. In [45], the extracted plate area is binarized and labelled to get the numbers. To identify the labeled segment as a number, several label layout patterns were used. The overall recognition rate of the system was 99%. In [47], a hybrid of connected components and blob coloring techniques was considered for segmentation. The overall accuracy of their system was 93.7%.

### 3.2. NP Segmentation Using Vertical/Horizontal Projection

A number plate has different colors for characters and background. The resulting binary image has different values for both the character and background of the number plate. In [19], pixel projection in vertical and horizontal directions is used for character segmentation. Projection techniques are used in [46,48,58,86,104,105,106,107,108,109,110,111]. To analyze the start and end points of characters, vertical projection is applied to the binary number plate, followed by horizontal projection, in order to extract individual characters. Apart from noise removal analysis of character sequence, vertical projection is applied for character extraction in [104]. This technique reaches up to an accuracy of 99.2% with processing speed of 10–20 ms for over 30,000 images. Profile projection method used in [108] is tested on a database of 560 images. The segmentation rate of 95.4% was achieved with an efficiency of recognizing multiple number plates present in single images. From review, it can be concluded that the technique following both horizontal and vertical pixel projections is the simplest and commonly implemented. The projection techniques show promising results for segmentation of characters as it is not dependent on their position. However, it requires prior knowledge of the character count. Image quality and noise may affect the projection values.

### 3.3. NP Segmentation Using Characters Features

Primary knowledge regarding characters facilitates the vehicle plate segmentation process. In [112] character isolation is done using the RGB color extractor. The segmentation rate is 98.5% for 255 color images evaluated. YOLO models, YOLOv2, Fast-YOLO, and Classification-Regression Network (CR-NET) [93], which are based on neural networks, are applied for segmentation in [78]. In [28], to recognize the locations of the starting and ending characters, the binary image is scanned horizontally. If the character-pixel to background-pixel ratio surpasses the threshold level just after being below the threshold value, this is termed as beginning point of the character. Paliy et al. [113], proposed resizing of the extracted vehicle plate into a known template proportion. All the positions of the characters in this template are known. Once resizing is done, the same locations serve as the characters. This technique is known to be simple. However, in case of extracted number plate shifting, it results in background rather than characters. One possible solution for severely damaged number plates is proposed in [114]. The color combination is utilized for locating the vehicle plate from the image. Each of the character dimensions is considered for character segmentation. Chinese number plate layout is used in the construction of recognition classifier. In [115], Taiwanese vehicle plates have similar color distribution with a white background and black characters. The number of color transitions from white to black or vice versa is maximum 14 and minimum 6 when the number plate is scanned horizontally. To rectify the rotation problem, Hough transform is applied. Hybrid binarization method is applied for character segmentation of dirty number plates. Lastly, the feedback procedure is implemented to manage the parameters. Around 332 distinct images are captured at varied distances and under different illuminations for their experiments. The overall rate of segmentation and localization are 96.4% and 97.1% respectively.

### 3.4. NP Segmentation Using Boundary Information

Character segmentation can also be achieved by modeling contours. Vertical edge detection with the long edge removal is used in [116,117]. Closed curved technique is used in [39]. In [36], vertical histogram is used for character segmentation. In [118], the segmentation process based on an adaptive morphology approach, for extracting severely degraded number plates, is proposed. Histogram related algorithm determines the sections and merges them. In [119], the algorithm of morphological thickening is used to find the reference lines to isolate the overlapped characters from each other. The algorithm of morphological thinning detects the baseline for connected character segmentation. About 1005 images were correctly segmented from an image set of 1189 degraded images, with a segmentation rate of 84.5%. In [120], a technique was demonstrated specifically for segmentation of the numerical characters on a number plate. This is done with the utilization of some dynamic programming. This method works at a quick pace by utilizing the algorithm’s bottom-up approach. Moreover, this method functions robustly by reducing the features that depends on the environment like edges and color. The rate of success of identifying main numbers is 97.14%.

## 4. Number Plate Recognition Methods

The final stage of ANPR systems is to recognize the segmented characters. The segmented characters may have different size and thickness due to zooming and camera distance [99]. The characters may be broken, tilted or effected by noise. Character recognition methods are covered in this section.

### 4.1. Character Recognition Using Template Matching

The simplest method for character recognition is Template Matching. It is a cross-correlation method in which the similarity of the extracted character is compared or measured with the template characters set. The character having the highest match with one of the candidates from within the templates set is chosen. The change in lighting condition directly affects the gray level intensities in the resultant image and for this reason, these methods are commonly used for binary images.

Template Matching Technique is used to recognize the segmented characters in [39], with success rate for four different sets of Moroccan format number plates as 98.1%, 96.37%, 93.07%, and 92.52% respectively. The use of template matching technique is also presented in [19,26,27,31,45,48,102,112,121]. There are many similar methods available in the literature such as Bayes decision [45], Jaccard Technique [48], Hamming distance [31] and Hausdorff Distance [122]. Normalized cross correlation is used for character recognition in [123]. The extracted characters are matched with the templates by scanning them column wise. The highest correlation value shows the best matched character. However, recognition by Template Matching is only successful if the characters are not broken, not tilted, have no fonts change, and have been resized to a fixed size [97]. To address this issue [124] uses several templates stored for a single character including tilt factor considering multiple orientations.

In [117], over 1200 images with dimensions 250 pixels wide, captured under various conditions and colors, had high extraction rate of 100% and reasonable recognition rate of 90%. In [125], over 1300 images of size 640 × 480 pixels with defined aspect ratio of license plate, are employed under various real time conditions. The extraction rate is efficient, almost 98.35%, while overall system performance for recognition is 92.12%, with processing time of 1.2 s at 10 FPS.

### 4.2. Character Recognition Using Extracted Features

Template matching technique involves all pixels which adds up to the processing time. The alternate method of feature extraction is also used for Character Recognition; it reduces processing time and eliminates the pixels of less importance [97]. SVM is used for character recognition in [126,127]. For challenging and complicated number plates, an improved SVM based algorithm is proposed in [128]. In [109,129], the features vector is produced by vertical and horizontal binary character projection. Each of the projections has undergone quantization into four specific levels, in [129]. In [109], Hoteling transformation is implemented on each character to produce the characteristic vector. This transformation is quite responsive to the segmentation output. In [47], division of binary character into 3 × 4 pixels’ block is done, a total of 24 blocks and each with a separate value that results in the generation of a characteristic vector. Each matrix is matched to a pre-defined template of characters set and as per template matching routine the closest matched character is selected for each block. After the characters’ identification, two separate groups of characters are formed and tested further using trained neural networks for improving the system recognition accuracy. Euler number and positioning check is performed for further enhancement of recognition rate. This technique took an average of 2.41 s to identify a Botswana number plate format and was unable to work well with vowels, angled/skewed and occluded/obscured plates. In [102], the process is quite similar to the previous, the only difference is in counting the elements having some degrees of inclination of 0, 45, 90, and 135. In [130], features scanning is implemented along the axis, which is located centrally. This axis links the horizontally bound lower and upper central moment. Then the transition between the number plate characters and the background as well as the gap between them forms a characteristic vector for individual character. This technique is constant in terms of character rotation. In [131], the characteristic vector is produced by demonstrating overall character contouring. The output waveform is passed through quantization to get the feature vector. This technique identifies variable sized and multi-font characters as the character contour does not change with the change in any font or size. In [132], character extraction is implemented by using Gabor filter. The character edges having the same orientation angle as the filter will have highest filter response. It can be utilized to generate characteristic vector per character. In [133], to extract characters in different directions from the character image, Kirsch edge detection is applied. This detection method for character identification and extraction yielded better acceptable results than the other techniques of edge detection including Wallis, Prewitt, and Frei Chen [134]. In [135], the characteristic vector extraction is done from binary image, followed by thinning operation to turn the character strokes direction into a singular code. In [136], the grey level values of the pixels of 11 sub-blocks are applied into the classifier of neural network as the characters. In [137], a scene is examined by reaching the non-overlapped 5 × 5 pixels blocks, thereby processing the overall image details to extract “spread” edge characteristics as per the experiment conducted in [138]. While in [139], following the coarse-to-fine recognition approach, the sub-image classification is described. In [71], three characteristic parameters, namely: peripheral background area, contour-crossing and directional counts are utilized where an SVM is used to perceive the classification.

## 5. Discussion

Some ANPR systems may use simple image processing techniques, performed under controlled conditions for predictable license plate styles. However, dedicated object detectors-such as HOG, CNN, SVM and YOLO to name a few-are used by advanced ANPR systems. Further advanced and intelligent ANPR systems utilize state-of-the-art ANPR software based on Neural Network techniques with AI capabilities. Just like many other fields, computer vision and machine learning have applications in ANPR too. The sheer diversity of license plate types across territories, states and countries makes ANPR challenging. The fact that any ANPR algorithm will need to work in real time further complicates Number Plate identification. Hence, utilizing ML, CV, AI techniques can relevantly empower ANPR.

Previous reviews from Literature are presented in Table 1. The cited works reviewed various techniques for each stage of ANPR system. The authors in [17] provided a good references collection for the new researchers in license plate detection field. However, the research did not compare the performance of recognition techniques in terms of accuracy rates.

The authors in [18] discussed the performance of the various algorithms used in the past. The percentage efficiency of the ANPR system per year was presented for years 1999–2015. The conclusions included that the efficiency of the ANPR system is not stable, and the performance varies due to various factors affecting ANPR like noise, environmental condition, choice for the algorithms and models training.

Table 2 presents the performance summary of various algorithms applied by researchers in the ANPR field, for different stages involved. Since this a multi stage/step algorithms, the only common parameter is the success rate/overall performance at each individual stage. The stages involved are shown in Figure 2. All the parameters in Table 2 heading were kept common for evaluating different approaches from researchers. The techniques listed from different researches under these header parameters is uncommon since this technology has no common algorithm to follow and it totally depends on the researchers that they mix and match various available algorithms and techniques to create their proposed recognition model for ANPR systems. The general input in such systems as given is the vehicle visual/image and is given as “datasets”, while output to the system is given as percentage of “overall recognition rate” that is the successfully recognized vehicle number plates in form of text string. This recognized data is then used for required post processing operations. Some of these stages use ML techniques. Since there is no uniform procedure available for ANPR systems for a side-by-side comparison, we have analyzed and summarized different algorithms that worked best with a variation of techniques applied. It is worth mentioning that the performance parameters we considered were constant for all, as shown in the table. We studied the procedures for all the studies/works presented in the Table 2; these included the general steps of number plate extraction, segmentation and recognition, and their success rates. Other factors considered for comparison are database size, image conditions, number plate formats, processing time, recognition rate, the device configuration for carrying out the test either in real time or on pre-acquired number plate images sets. The potential issues or problem areas, where identified by the researchers, are also discussed. From this analysis, we can conclude that the algorithms presented for the real time systems showed great overall accuracy rate, which is very impressive. However, uniform evaluation of different algorithms has requirements as indicated towards the end of the Conclusion Section.

In [24], 99.5% accuracy rate is achieved by using HD cameras for over 2790 characters tested in real time. CCA with OCR algorithms were used for Qatar number plates format. However, it is clearly computationally intensive, and involves high system cost with memory and processing time constraints. In [78], the number plate extraction rate is 98.33% to 100% for different datasets tested in real time scenarios for Brazilian number plates. The overall recognition efficiency is 93.53% (dataset SSIG) and 78.33% (UFPR-ALPR). The authors here applied the CNN based algorithms and YOLO object detector. In [142], scale adaptive model is tested for real time scenarios with an overall success rate of 97% by applying it to over 2600 mix format number plates. However, the process required extensive model training to cover varying situations. The Local Binary pattern extraction with Tesseract’s OCR is applied in [125] for real time scenarios with tested dataset of about 1300 images. The overall accuracy is 96.73% is impressive but the fact that the authors only considered fixed angles for image acquisition and hence there is still room to research different angles with same algorithms applied.

A rough number plate condition may affect the process of recognition and the environmental conditions makes it even more challenging. Current ANPR systems use low resolution camera most of the times to keep the system cost lower and hence the video quality in real time has enough probability to make errors. HD cameras can be used to solve this issue as these cameras have the capacity to capture fine details from even long ranges. ANPR accuracy can be improved considerably if the camera is correctly setup by keeping the distance, tilt angles, region of interest zooming and lighting factors in consideration. Depending on the environment and camera shutter speed, the processing capabilities vary. In [26], HD cameras are employed. The recognition rate of 98% is achieved. In [24], the recognition rate of similar system is 99.5%. However, systems with HD cameras are computationally expensive, involve higher cost, greater memory requirements and also have processing time constraints. Some studies involved the use of classifiers for the recognition purpose. They are either using a combination of multiple classifiers in parallel or have multistage classification schemes. Artificial Neural Network is used in [126]. Convolutional Neural Networks is used in [78] in real time scenario and has shown great results for each stage of ANPR system. Neural network based algorithms seems promising for ANPR and are proposed in [19,103,145,146,147,148,149,150,151,152,153,154,155,156,157,158,159,160,161,162].

In [163], an alternative but unique self-learning algorithm, based on Bayesian-probability and Levenshtein-Text-Mining, is proposed. It offers higher matching accuracy of ANPR system. It utilizes conditional probabilities of observing one character at a station for an assigned character at some other station, using the “Association Matrix”.

Tesseract is the most widely adopted OCR engine, with the ability to recognize over 100 languages and is not limited to further training of new/unlisted languages. Majority of the AI/ML based ANPR software providers utilize this engine for vehicle recognition applications. There are many vendors providing ANPR solutions around the globe. For example, the OpenALPR specializes in license plate and vehicle recognition software. It is an open-source license plate reader service provider and is available as commercial software too. The most convenient feature is the compatibility with most cameras and diverse environmental conditions. Since this is based on artificial intelligence and machine vision technology, its success is solely based on the effectiveness of the algorithms used in the software along the hardware employed in the ANPR system.

Public and commercial vehicle image-datasets are available for testing ANPR algorithms and some of these are listed in Table 3. These datasets are of great help to the research community and are widely utilized with attributions to the providing source. Researchers can use these images for testing the accuracy of their algorithms. These datasets comprise of a great number of real-time images captured under diverse conditions. These images/videos have variations in backgrounds, lighting conditions, environmental conditions, plate position, physical conditions, size, style format and possible real time effecting metrics. Some of them have both open-source and commercial versions available; the latter normally using different algorithms for OCR, based on larger datasets, to offer higher accuracy than the former.

There are quite many vendors serving the ANPR solutions systems but not all of them provide the same services and user feasibility with relative concerns. Selecting the right software is the key to getting the required accuracy in the recognition process and avoiding unwanted circumstances of incorrect identification of the number plate or no recognition at all due to scene complexity or ineffective algorithms used. To choose the better performing ANPR system, one must evaluate the software providers based on the criteria of pricing, working environment, developer support from the vendor, free trials and data accuracy tests.

## 6. Conclusions and Future Research

This paper presents a detailed survey on ANPR algorithms proposed and experimented in recent relevant studies. We categorized these algorithms according to the features required in recognition process at individual stages. Each stage is presented in detail for performance summary along with issues and challenges, where applicable. However, it is difficult to have a uniform evaluation and comparison if the dataset is not common, as explained later.

ANPR systems are based on complicated optical, computing and digitizing capabilities that may result in a slow recognition process of plates. The ANPR solutions available in the market do not offer a standardized set for all the countries; each company has to be provided with a well optimized system for different parts/regions of the world, since the same system as developed is not sufficient and needs to be designed according to the region where deployed; keeping all the affecting factors in considerations. OCR engines often are optimized for specific countries. It needs to be made sure if the required countries are supported in the library or engine that is installed on the camera. Each ANPR solutions system provided by vendors has its own strengths and weaknesses. The best among these is the one that caters for the needs of the region in identified system effecting conditions of that area.

Future research in ANPR still faces several challenges; For instance, there is a need to concentrate on more robust algorithms for non-standardized formats, irrespective of regions. Also, all proposed/designed algorithms need to be tested for real time scenarios rather pre-acquired images. In addition, high resolution cameras need to be integrated, allowing robust algorithms to reduce processing times and increase recognition capabilities. Yet another avenue is Obscure character recognition, since there are a lot similarities in characters like the pairs-(O,0), (P,B), (Z,2), (S,5), (3,8), (B,8), (P,R), (D,O), (1,I), (Q,O), (C,G), (A,4), (K,X), (F,E), (b,6), (q,9), (p,b), (V,W), (X,Y), (V,U), (6,8), (5,3), (5,8), (0,8), (3,9), (4,9) etc. The similarities, together with impairments, can easily deceive the optical character recognition mechanism if there is small tilt, fonts change, broken, snow or dirt on characters or if the image is acquired at different angles. Lastly, it is recommended that moving vehicles, fast speeds, low contrast, insufficient or over exposed lights and real time scenarios must be tested to check robustness of the proposed algorithms.

With recent advancements in Deep learning, Computer vision systems are enabling numerous exciting applications, ranging from safe autonomous driving to accurate object recognition, to automatic reading of images in various applications [174]. Other real time object detectors such as YOLO, can be trained and evaluated for this system [175].

Android platform has gained much importance in the technology field and numerous applications are being integrated with it. Many researchers have proposed the ANPR systems built on android platforms. However, their performance is very limited and have several constraints that can be worked on in future to develop a comparatively accurate phone-based recognition system for vehicles. Future concerns are memory resources, use of Global Positioning System coordinates for geo-tagging and online databases for respective applications [143].

Other than image processing based ANPR systems, RFID based vehicle verification systems are also emerging and being used in many countries for transportation applications. Radio Frequency Identification based vehicle recognition is another way to recognize vehicles identity or track them. Its purpose on road applications is similar to image processing based ANPR recognition system but the working terminology is different. RFID technology is proven to provide an effective solution to different tracking and localization problems that are more common in Image processing based systems. The most important step involved in recognition within CS/ML based ANPR systems is the extraction of number plate from the scene, which is most complex part in terms of performance. While using RFID for extraction/identification purposes, in case of missed vehicles this technology may come in action hence helping the ANPR. Also, the speed detection can be performed with RFIDs techniques. The vehicle may be tracked with RFID technology irrespective of its location whether is it within or without line of sight to the camera. The vehicle can be easily tracked throughout its travel on the road depending upon the types of RFID technology utilized. RFID allows toll payments facility as well. In short, RFID works on radio frequency whereas the image processing based ANPR systems are dependent on camera. RFID does not require any camera and it can communicate with the tag on the vehicle on the go, eliminating many complexities that are associated with camera dependent technologies.

Image processing based ANPR integration with RFID technology may help in various road applications and may improve system efficiency as in [176,177]. The integration of RFID and ANPR may result in a hybrid system and it can be considered for multiple applications of intelligent transport systems in present and future [176].

It is important to mention RFID technology here as future technology since many countries are now considering the integration of ANPR and RFID to take maximum benefit of the hybrid solution making the transportation system more accurate and secure. Both systems have their strengths and weaknesses. For ANPR, in terms of algorithms and performance, the main weakness is the successful localization of the vehicle number plate which is very much dependent on the camera and many other factors that makes it challenging to successfully recognize a vehicle number plate in some conditions mentioned earlier. Also, no additional transponders or tags are required to attach on the vehicles. The strength of this technology is that along with the recognition of vehicle its very helpful for security/surveillance applications since it captures the vehicles visuals.

The strength of RFID is the highest accuracy rates for recognition since it works on radio frequency detections by sensing the transponder attached on the vehicle, in most cases a label or tag. It can track the vehicle throughout the travel irrespective of the line of light as compared to the cameras based systems. It can effectively be used in etoll collection and the tag data can be updated accordingly. The weaknesses to this technology is limited in case of recognition/reading vehicle however it does not store any visuals of the scene. Detection accuracy from RFID and visual security sense from ANPR together creates a hybrid system which can make the transportation system more secure and accurate.

This technology is unfortunately not a one-size-fits-all solution and needs optimization from region-to-region. To allow a uniform evaluation of different approaches, the proposed algorithms needs to be tested using complex datasets provided various factors as diversity in number plate styles, colors, fonts, sizes, orientations/tilt/skewed, occlusions, obscure characters and other physical conditions, camera resolution, shutter speed, lightening/illumination aids, coverage capability for number plate extraction from the real time complex scenes, fast moving vehicles and to maintain low processing times and increase recognition capabilities in real time scenarios. A real-time video scene is recommended for the tests rather than using pre-taken still images.

The current state of the art approaches are more inclined towards the use of OCR engines equipped with AI capabilities. Recognition algorithms based on Artificial Neural Networks are providing better recognition rates. Integration of the ANPR system with other ICT tools is also gaining popularity such as integration of ANPR engines with GPS, Online databases, Android/IOS platforms, RFID and other various tools that serves different applications in intelligent transportation systems. Future research is needed to highlight importance and ways of incorporating this technology with other ICT tools which can be beneficial for the transport system and its policy making. The available CV algorithm’s accuracy is limited to particular regions and its standardization for the number plates. Further research is needed to make the algorithms smart enough to work in variable environments provided non-standardized diverse number-plate datasets.

Now that the accuracy of ANPR systems is improving with time and are being used in tandem with AI capabilities and IoT, it is expected that these disruptive technologies and applications will be more widely adopted and that new use cases will emerge in the coming times. It is possible with the relevant tools/software to transform the raw augmented ANPR camera data into practical knowledge and help understand the traffic flow including passenger and freight mobility. ANPR cameras, have the potential and can be augmented with vehicle category information [178].

## Figures and Tables

**Table 1:** Previous Reviews from Literature.

Year	From	Techniques Reviewed
2018	[16]	Reviewed Myanmar Researched Papers Only.
		Khin et al. in the year 2018 - Threshold based approach and bounding box - All colors, and conditions images are used
		Htay et al. in the year 2016 - Neural Network Method for Plate Localization - Noisy, tilted plates or other degraded forms of number plate were not considered em - Very limited number of samples tested
		Sinha et al. in the year 2011 - Character extraction and recognition - Pattern recognition based on fuzzy logic - Did not yield good results for skewed plate
		Bai et al. in the year 2004 - Hybrid Approach - Edge detection and morphological operations - Tilted/skewed characters are not tested
ine 2017	[17]	This research reviewed various techniques for each stage of ANPR system.
		License Plate Extraction: - Edge information Analysis - Probabilistic model - Subspace Projection and Probabilistic Neural Network - Blob Analysis, Mathematical Morphology - Color Space and Geometrical Properties - Thresholding, Histogram, Computational Intelligence and Adaptive Boost techniques
		Segmentation: - Gabor transform - K-Means Algorithm - Tree of Shapes - Hidden Markov Chains
		Recognition: - Optical Character Recognition (OCR) - Embedded DSP-Platform - Pattern match method - Computational Intelligence - Neural Networks
2016	[18]	Reviewed techniques:
		Feature salience, Hough Transform, Neural Networks, Histogram techniques, Chin code, Tophat Filtering, Template matching, Matrix mapping, wavelet transform, SVM, Pulse Coupled Neural X Networks, SIFT feature points, Gaussian Filtering and other modified NNs.
2015	[19]	Techniques reviewed for each stage:
		Number Plate Extraction - Method 1 * Sobel based Vertical Edge Detector * Sliding window technique, it has the size similar to that of the number plate size * Requires prior knowledge of the number plate size * Threshold tuning required and needs to setup for each set - Method 2 * Gradient analysis * Vertical histogram along with morphology techniques * Connected Component Analysis - Method 3 * Geometrical shape of the number plate
		Character Segmentation Method - Pixel projection in which both vertical and horizontal directions employed - Fast and robust - Dealt with tilt factor by adding additional layer of vertical projection
		Character Recognition Techniques - Method 1 * Template Matching * Adaptive threshold * Pixel-wise matching is performed * The simplest method known for recognition - Method 2 * Neural Networks classifiers
		The performance of these techniques reviewed is summarized in Table 2.

**Table 2:** Performance Summary of ANPR system techniques.

No.	From		Procedure		Database	Image Condition	Extraction Rate	Segmentation Rate	Recognition Rate	Overall Recognition Rate	Processing Time	Real Time	Device Config.	Plate Format	Problem Areas
		Extraction	Segmentation	Recognition											
1	[39]	Otsu Adaptive thresholding, CCA, Edge Detection-Canny	Closed curves	Template Matching	Set 1: 533 Set 2: 651 Set 3: 757 Set 4: 611 (Video Sequences)	Various situations with different Light Conditions	Set 1: 96.37% Set 2–4: 96.06%	—	Set 1: 98.1% Set 2: 96.37% Set 3: 93.07% Set 4: 92.52%	—	—	No	4GB memory DDR4 and 3.4 GHz Intel(R) Core(TM) i5 CPU - MATLAB R2015b	Moroccan, four different formats	—
2	[38]	Edge statistics and morphology techniques	Bounding box	Template Matching	9745 images	—	98%	—	82.6%	75–85%	—	No	MATLAB	Indian	Not suitable for different orientations.
3	[140]	Used provided Images	—	HOG feature and Extreme Learning Machine	69 images, 45 images used as trainers, 5 classes	Low resolution portion of the image, 15–18 px height	—	—	90%	90%	—	Yes	—	South Thailand	Day time only, no license localization process is applied.
4	[141]	Morphology Techniques	Region props bounding box using Matlab	Template Matching	30 images	Low brightness, contrast	92%	97%	98%	98%	—	No	Matlab	Multi Fonts, Indian	—
5	[36]	Histogram Analysis using HOG	Vertical Histogram	OCR – Template Matching	110 images	Various Conditions	89.7%	—	—	—	—	No	OpenALPR	Europe	Cannot detect beyond 30-degrees horizontal/vertical angle, if the car is moving (image blur) or there is low light
6	[78]	Object detection, CNNs—(YOLO Detector)	Character Segmentation CNNs, Bounding box	Data augmentation, Distant CNNs for letter and Digits	SSIG Dataset: 2000 Frames, UFPR-ALPR: 4500 Frames	1920 × 1080 pixels	SSIG: 100.00% UFPR-ALPR: 98.33%	SSIG: 97.75% UFPR-ALPR: 95.97%	SSIG: 97.83% UFPR-ALPR: 90.37%	SSIG: 93.53% UFPR-ALPR: 78.33%	SSIG: 21.31 ms, 47 FPS UFPR-ALPR: 28.30 ms, 35 FPS	Yes	NVIDIA Titan XP GPU (3840 CUDA cores and 12 GB of RAM	Brazil	Adjustments have to be made for other than Brazilian formats. Dependent on license plate layout.
7	[125]	Cascade classifier with LBP features (Local Binary Pattern)	—	Tesseract’s OCR	1300 images	640 × 480 pixels with 50 × 11pixels aspect ratio of license plate, various conditions	98.35%	—	92.12%	96.73%	1.2 s,10 FPS	Yes	RaspberryPi 3 Model B operating at 1.2 GHz with 1 GB RAM	Indian	Dependent on standardization for detection too, overall accuracy is for front side license plate only at fixed 90d angle, High processing time.
8	[117]	LBP, Character and edge information	Vertical Histogram	Tesseract OCR with preprocessing techniques	1200+ images	250 pixels wide, Various conditions and colors	100%	—	90%	90%	Vary	No	OpenALPR	Myanmar	High processing time.
9	[23]	Vertical and horizontal edge histograms	Connected component labeling and morphological method	Statistical features matched with stored ones	50 Images	Variable size and style, Latin script only	90%	91%	93%	92.75%	—	No	MATLAB R2015a	Pakistan	Very Limited dataset tested
10	[26]	Image resizing using nearest neighbor interpolation, Preprocessing and geometrical conditions	CCA labelling and morphological operations	OCR algorithms	958 images	HD images, various conditions	98.10%	99.75%	99.50%	98%	61ms	No	MATLAB	Qatar	Computationally intensive cost, HD camera used—memory and time constraints
11	[121]	—	Preprocessing techniques, Otsu’s Thresholding	Bounding box feature and template matching OCR	14 images	8 mp camera, different timings and distances	—	—	—	92.85%	—	No	MATLAB	Malaysia	Limited set of images, cannot recognize low quality images, works for standardized format only
12	[25]	Geometrical features using Mathematical Morphology	—	—	571 images, multiple sets	Complex images, 1792 × 1312, 800 × 600, and 640 × 480 pixels	—	—	—	98.45%	20 ms	No	MATLAB 2.7 GHz core i7, 8 GB of RAM, Python Raspberry Pi with 700 MHz processor and 256 MB of RAM	Greek	Higher processing time, cost and power for higher resolution images
13	[103]	ROI extraction using intensity detection and morphological operations	Otsu’s Thresholding with preprocessing techniques	OCR with Correlations approach	40 Images	480 × 640 pixels	87.5%	—	85.7%	86.6%	—	No	MATLAB R2014a.	Iraq	Failed for multiple objects in the scene and for unclear images or algorithm removing objects by mistake
14	[142]	Scale-Adaptive System, Feature computation with Gentleboost algorithms	Scale-weighted linear interpolation	Scale-adaptive model and empirically constrained-deformation model	2600+ images Multiple Datasets OS, Stills&Caltech, AOLP	Variable Distances between camera and vehicle, scenes and sizes (in color JPEG format)	OS: 87.38% Stills&Caltech: 84.41%	OS: 74.29% Stills&Caltech: 84.13%	98.98%	97%	3.16–9.43 s	Yes	PASCAL Visual Object Classes	USA, Taiwan, Spanish	Extensive training required comprise all the possible situations, Segmentation process can be enhanced by using additional morphological techniques.
15	[126]	Support Vector Machine (SVM) with Preprocessing techniques	Threshold, Morphological operations and Contours Algorithms	Artificial Neural Network (ANN)	—	—	—	—	—	—	—	No	Intel core i5 PC, C++ with OPENCV 3.2.0 Library	Spain	—
16	[24]	Image resizing, Preprocessing and geometrical conditions	CCA labelling and morphological operations	OCR using Field Programmable Gate Array (FPGA) Processing Unit	454+ Images, 2790 Characters	HD images with 34 × 22 character size matrix, various light and weather conditions	—	—	—	99.50%	3.78 ms	Yes	Matlab, Xilinx Zynq-7000 All Programmable SoC, FPGA/ARM	Qatar	Computationally intensive cost, HD camera used-memory and time constraints
17	[116]	Region of Interest (ROI) based filtering	Vertical Edge Detection with removal of long edges	—	1000+ videos	Different orientation, light conditions and type of vehicles	—	—	—	92.31%	8.3 FPS	Yes	—	Indian	Format dependent
18	[103]	Intensity detection and mathematical morphological operations	Labeling connected components	Back propagation Neural Network (BPNN)	60 images	Variable size and illumination condition	98.3%	—	93.2%	97.75%	—	No	MALAB R2014a	Iraq	—
19	[19]	Method E1: Vertical edge detection using Sobel filter Method E2: Gradient extraction, vertical histograms, CCA Method E3: Using shape features	Pixel Projection in Vertical and Horizontal directions	OCR1: Template Matching OCR2: PNN	141 images	1024 × 768 pixels, Variable conditions	E1: 65.25% E2: 43.26% E3: 33.33%	E1: 60.87% E2: 63.93% E3: 65.91%	OCR1 for E1: 81.99% E2: 78.65% E3: 81.50% OCR2 for E1: 82.42% E2: 78.36% E3: 77.95%	OCR1 for E1: 42.41% E2: 28.12% E3: 21.66% OCR2 for E1: 42.01% E2: 27.86% E3: 21.46%	—	No	—	Canada	All methods were tested as from literature to verify the researcher’s claims and almost all failed for variable datasets.
20	[112]	Pixel Statistics with pre-processing techniques	RGB Color Extractor and character isolation using thresholding.	Template Matching	255 Images	Color Images, 2448 * 3264 * 80 pixels Camera: iPhone 5s (Variable light conditions)	—	98.5%	95.1%	95%	—	Yes	Tesseract-Open-source OCR engine	United States (Illinois)	Format specific. Not compatible with low light images. Ambiguous characters have low recognition
21	[143]	Filtering techniques with contrast enhancement and other preprocessing techniques	Vertical Projection method	OCR using ANN	—	Color Images taken with 5MP phone built-in Camera, 1600 * 1200 pixels	—	83.5%	92%	88%	—	Yes	Eclipse IDE, Android Platform SDK Processor: ARM v6 800MHz RAM: 285 Mega Bytes Screen Size: 320 × 480 Camera: 5 Mega Pixels OS Version: 2.3 Ginger Bread	Malaysia	Low resolution camera, Format specific, Limited system memory, Motion blur, object obscuring, day/night shots are problem areas for the system. It can be improved a lot in future.
22	[144]	Color features (Hue and shape) with vertical/slope sweep	Image Processing Filter (Histogram, Laplacian, Morphology), Connected Component/Projection Analysis	Decision Tree and SVM	Set1: 1150 Images Set2: 540 Images	Set1: Gatso control speed cameras on highways Set2: Parked Vehicles captured with phone camera 1.3MP. (Different conditions)	Set1: Speed Lane: 96.6% Mid Lane: 93.14% Side Lane: 78.8% Multi Vehicles: 96% Set2: Day: 96.8% Night: 91.4% Angled upto 20°: 74.6%	—	—	Speed Lane: 92.6% Mid Lane: 87.14% Side Lane: 64.8% Multi Vehicles: 94% Set2: Day: 94.4% Night: 72.14% Angled upto 20°: 62%	0.75–1.59 s (Total system response)	Yes	1.7 GHz CPU with 4 GB RAM	Iran	Practical and accurate for targeted lane. The claimed 96%, 94% system performance is tested on a very limited data set. The overall performance considering other lanes is comparatively poor in recognition for set1. In set2, only daylight images are well recognized with poor results otherwise.

**Table 3:** ANPR Datasets available to the research community.

Source/API	Datasets	Size (1 k = 1000)	Version and Availability ${}^{2}$
[79]	Sighthound	Over 3 million images	Open source Commercial Publicly available to the research community
[164]	ImageNet	Over 14 million images	Publicly Available
[78]	UFPR-ALPR	4.5 k images	Non-commercial use only Available on request for academic purposes
[165]	CompCars	136.7 k images	Non-commercial Research purposes only
[166]	CCPD: Chinese City Parking Dataset	250 k unique images	Open source under MIT license
[167]	VMMR: Vehicle Make and Model Recognition	291.7 k images	Publicly available to the research community Latest versions of dataset can be provided upon request
[168]	SSIG-SegPlate	6.6 k images	Available on request only
[169]	AOLP: Application-Oriented License Plate Recognition	2 k images	Available on request for research purposes only
[170]	Cars Dataset by Stanford	16.1 k Images	Publicly available for research purpose only
[171]	Cityscapes Dataset	25 k Images	Non-commercial Research purposes only
[172]	Caltech	1.5 k images	Available with attributions
[173]	GTI: Grupo de Tratamiento de Imágenes	3.4 k with vehicles while 3.9 k without vehicles in scene, 4000 from other sources	Publicly Available

## Data Availability

Not applicable.
